# Correction to: For antibody sequence generative modeling, mixture models may be all you need

**DOI:** 10.1093/bioinformatics/btae407

**Published:** 2024-06-24

This is a correction to: Jonathan Parkinson, Wei Wang, For antibody sequence generative modeling, mixture models may be all you need, *Bioinformatics*, Volume 40, Issue 5, May 2024, btae278, https://doi.org/10.1093/bioinformatics/btae278

The published article has been updated to list Wei Wang as corresponding author.

